# Closing the gap: yeast electron‐transferring flavoprotein links the oxidation of d‐lactate and d‐α‐hydroxyglutarate to energy production via the respiratory chain

**DOI:** 10.1111/febs.14924

**Published:** 2019-05-25

**Authors:** Marina Toplak, Julia Brunner, Chaitanya R. Tabib, Peter Macheroux

**Affiliations:** ^1^ Institute of Biochemistry Graz University of Technology Austria

**Keywords:** d‐α‐hydroxyglutarate, electron‐transferring flavoprotein, enzyme kinetics, flavin adenine dinucleotide, *Saccharomyces cerevisiae*

## Abstract

Electron‐transferring flavoproteins (ETFs) have been found in all kingdoms of life, mostly assisting in shuttling electrons to the respiratory chain for ATP production. While the human (h) ETF has been studied in great detail, very little is known about the biochemical properties of the homologous protein in the model organism *Saccharomyces cerevisiae* (yETF). In view of the absence of client dehydrogenases, for example, the acyl‐CoA dehydrogenases involved in the β‐oxidation of fatty acids, d‐lactate dehydrogenase 2 (Dld2) appeared to be the only relevant enzyme that is serviced by yETF for electron transfer to the mitochondrial electron transport chain. However, this hypothesis was never tested experimentally. Here, we report the biochemical properties of yETF and Dld2 as well as the electron transfer reaction between the two proteins. Our study revealed that Dld2 oxidizes d‐α‐hydroxyglutarate more efficiently than d‐lactate exhibiting *k*_catapp_/*K*_Mapp_ values of 1200 ± 300 m ^−1^·s^−1^ and 11 ± 2 m ^−1^·s^−1^, respectively. As expected, substrate‐reduced Dld2 very slowly reacted with oxygen or the artificial electron acceptor 2,6‐dichlorophenol indophenol. However, photoreduced Dld2 was rapidly reoxidized by oxygen, suggesting that the reaction products, that is, α‐ketoglutarate and pyruvate, ‘lock’ the reduced enzyme in an unreactive state. Interestingly, however, we could demonstrate that substrate‐reduced Dld2 rapidly transfers electrons to yETF. Therefore, we conclude that the formation of a product‐reduced Dld2 complex suppresses electron transfer to dioxygen but favors the rapid reduction in yETF, thus preventing the loss of electrons and the generation of reactive oxygen species.

**Abbreviations**
2HG: d‐2‐hydroxyglutarate or d‐α‐hydroxyglutarate
DCPIP: 2,6‐dichlorophenol indophenol
Dld2: d‐lactate dehydrogenase 2 from *Saccharomyces cerevisiae*
ETF: electron‐transferring flavoprotein
ETF‐QO: ETF‐ubiquinone oxidoreductase
FAD: flavin adenine dinucleotide
hETF: ETF from *Homo sapiens*
PCMH: *p*‐cresol methylhydroxylase
yETF: ETF from *Saccharomyces cerevisiae*

## Introduction

Since the discovery of the first electron‐transferring flavoprotein (ETF) by Crane *et al*. 1 in 1956, a variety of other proteins belonging to the same family have been identified in all kingdoms of life. They are all heterodimeric proteins, consisting of a larger α‐ and a smaller β‐subunit and mostly bind a single flavin adenine dinucleotide (FAD) cofactor at the subunit interface 2, 3. In prokaryotes as well as eukaryotes most of them function as housekeeping proteins by coupling the degradation of fatty and amino acids (in humans also of choline) with energy production via the respiratory chain 2, 4, 5. This feature is based on the ability of ETFs to accept electrons from various dehydrogenases involved in catabolic pathways and the transfer to an integral membrane protein, ETF‐ubiquinone oxidoreductase (ETF‐QO), which in turn reduces ubiquinone, a key component of the respiratory chain, and thus directly influence the generation of ATP. In some cases, however, ETF is part of a specialized system, for example, in *Methylophilus methylotrophus* and *Peptostreptococcus elsdenii*. In these bacteria, reduced trimethylamine dehydrogenase and NADH, respectively, serve as the predominant source for electrons, as these organisms lack the metabolic pathways mentioned above and, therefore, rely on other carbon sources (methanol or trimethylamine and d‐lactate, respectively) for energy production 6, 7, 8, 9, 10. In other cases, reduced ETFs transfer electrons to acyl‐CoA dehydrogenases, which subsequently catalyze the reduction in short‐chain α,β‐unsaturated fatty acids 8. The cellular role of the electron‐transferring flavoprotein of *Saccharomyces cerevisiae* (yETF) appears to be similar to bacterial homologs because only one enzyme is currently suspected to deliver electrons, that is, d‐lactate dehydrogenase (Dld2), whereas yETF was reported to deliver the electrons to an ETF‐QO homolog, named Cir2p, and thus shares this feature with other eukaryotic organisms 11, 12.

The mitochondrial matrix protein Dld2 was first discovered in a yeast two‐hybrid assay, which was used to identify actin‐interacting proteins (therefore it was initially called Aip2) 13, 14, 15. In the course of subsequent *in vivo* characterizations, however, it was shown that the protein catalyzes the oxidation of d‐lactate to pyruvate. Interestingly, recent studies by Becker‐Kettern *et al*. 16 revealed that Dld2 was also able to oxidize d‐α‐hydroxyglutarate to α‐ketoglutarate. In fact, according to steady‐state experiments (with truncated Dld2) d‐α‐hydroxyglutarate was oxidized with a higher efficiency compared to d‐lactate 16. In any case, the oxidation of the two substrates results in the reduction in the flavin cofactor, which needs to be regenerated for turnover. Although the natural electron acceptor of reduced Dld2 remains elusive, the presence of a gene encoding an ETF suggested this protein to serve as the electron acceptor, which then further transfers the electrons to a quinone‐dependent dehydrogenase in the inner mitochondrial membrane (Cir2p).

To study the possible interaction and the electron transfer between Dld2 and yETF, we produced both proteins in *Komagataella phaffii* (formerly known as *Pichia pastoris*) and *Escherichia coli*, respectively. In a detailed biochemical characterization of yETF, we could show that it exhibits spectral and electrochemical properties strongly diverging from its human counterpart 17, despite high sequence identity (47%) and structural similarity. Because we identified only a single amino acid residue in the active site of yETF to be different in comparison with the human homolog (βPhe19 vs βTyr16), we asked the question whether the replacement of this particular phenylalanine by tyrosine could restore the biochemical properties of hETF 17, 18. Therefore, we also generated a βPhe19Tyr variant, as well as two double variants (βPhe19Tyr‐αAsn269Ala and βPhe19Tyr‐βGlu169Ala), with an additional amino acid exchange targeting the well‐studied salt bridge at the surface of the human protein (equivalent to human αAsn259Ala and βGlu165Ala variants, respectively) 19.

To better understand the catalytic properties of full‐length Dld2, we performed a detailed kinetic characterization of the enzyme involving steady‐state and presteady‐state experiments with both substrates, that is, d‐α‐hydroxyglutarate and d‐lactate. As previously reported by Becker‐Kettern *et al*. 16, steady‐state experiments revealed a higher catalytic efficiency and lower *K*_Mapp_ with d‐α‐hydroxyglutarate, but also a lower turnover. In our presteady‐state experiments, however, d‐α‐hydroxyglutarate was shown to reduce Dld2 about ten times faster than d‐lactate, which indicated that the oxidative half reaction was rate limiting under steady‐state conditions using 2,6‐dichlorophenol indophenol as electron acceptor. Finally, we could show that substrate‐reduced Dld2 rapidly transfers electrons to yETF, suggesting that product binding to the reduced Dld2 suppresses reaction with oxygen but not with yETF. Thus, our study revealed that (a) Dld2 is in fact a more efficient d‐α‐hydroxyglutaric acid dehydrogenase, (b) electron transfer is controlled by product binding to reduced Dld2 and (c) yETF is the natural electron acceptor of Dld2.

## Results

### Production, purification and biochemical characterization of yETF

**Figure 1:**
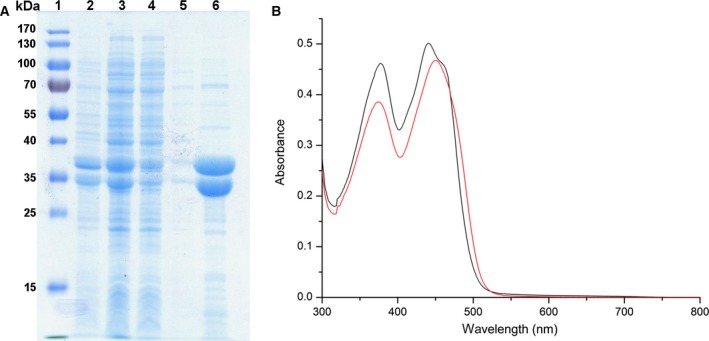
SDS/PAGE analysis of the different steps in yETF purification (A) and UV‐visible absorption spectrum of native (black line) and denatured (red line) yETF (B). (A) SDS/PAGE analysis of the different fractions collected during purification of yETF wild‐type using Ni‐nitrilotriacetic acid affinity chromatography. In lane 1 PageRuler^®^ prestained protein ladder (Thermo Fisher Scientific), in lane 2 the cell pellet after lysis, in lane 3 the cell lysate, in lane 4 the column flow through, in lane 5 the wash fraction and in lane 6 the elution fraction (α‐subunit of yETF at 38 kDa; β‐subunit at 37 kDa) is shown. (B) UV‐visible absorption spectra of yETF diluted to a final concentration of 40 μm with 50 mm HEPES pH 7 + 1 mm DTT (black line) and denatured using 2% SDS (red line) were recorded between 300 and 800 nm at 25 °C. Based on the extinction coefficient of free FAD (denatured, red line) at 450 nm (11 300 m ^−1^·cm^−1^), the extinction coefficient of yETF wild‐type at 450 nm (11 600 m ^−1^·cm^−1^) and 469 nm (9900 m ^−1^·cm^−1^) could be determined.

Recombinant production of the heterodimeric yETF in *E. coli* and subsequent purification using Ni‐nitrilotriacetic acid affinity chromatography yielded about 3–5 mg of pure heterodimeric protein (Fig. 1A, lane 6) per g wet cell weight, which allowed its detailed biochemical characterization. Like other flavoproteins yETF exhibits a characteristic UV‐visible absorption spectrum with maxima at 377 and 441 nm, which are slightly shifted upon denaturation (Fig. 1B, black and red line, respectively). The maxima at 373 and 447 nm observed in the absorption spectrum of denatured yETF further indicate the presence of an FAD chromophore, which is also present in all other ETFs studied so far 7, 18, 20, 21, 22, 23.

**Figure 2:**
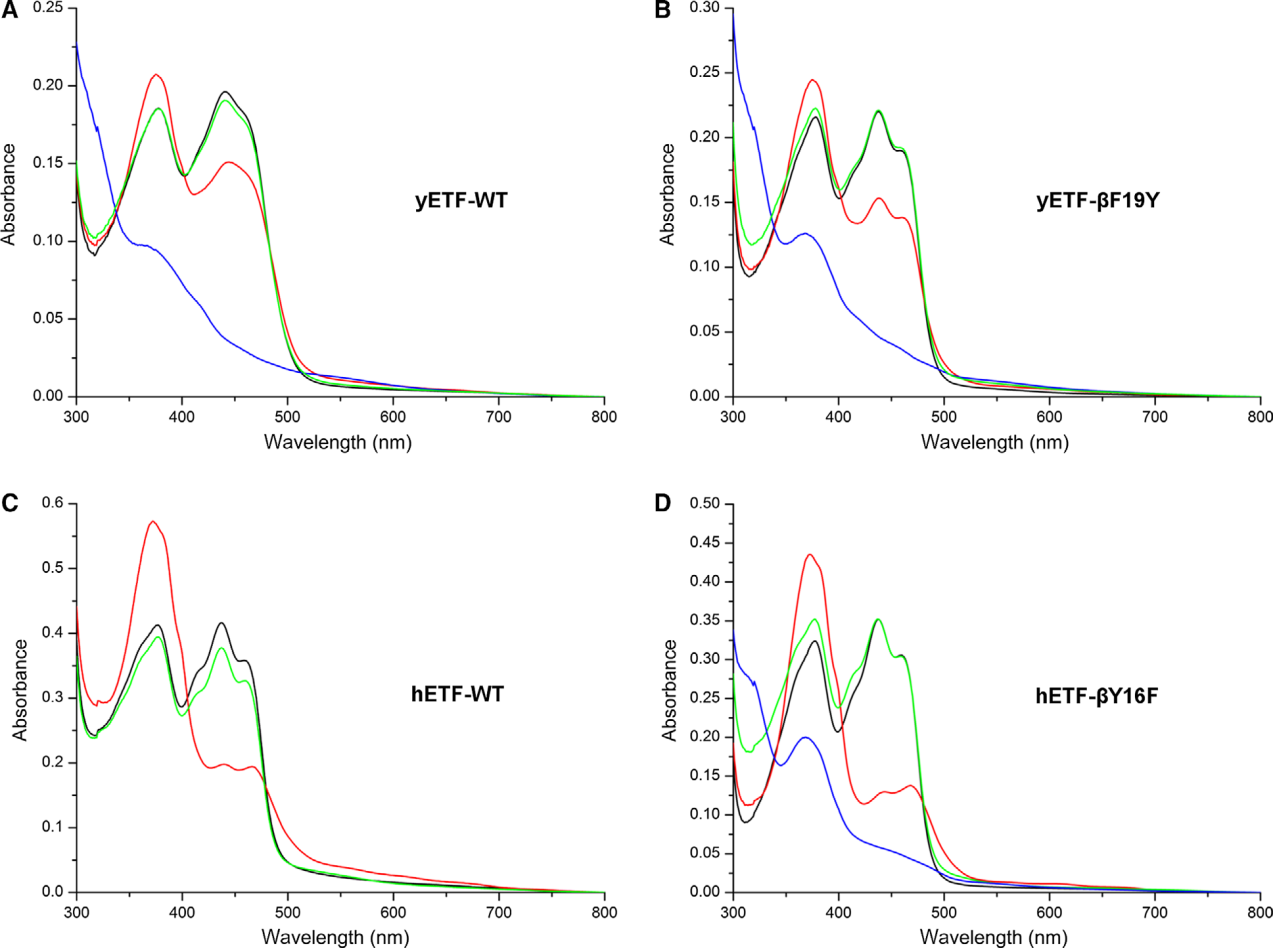
Photoreduction of yETF wild‐type (A) and yETF‐βPhe19Tyr (B) as well as of hETF wild‐type (C) and hETF‐βTyr16Phe (D) under anaerobic conditions. UV‐visible absorption spectra (300–800 nm) of yETF wild‐type and yETF‐βPhe19Tyr as well as of hETF wild‐type and hETF‐βTyr16Phe, diluted to a final concentration of 30 μm using 50 mm HEPES pH 7 containing 1 mm DTT, recorded during anaerobic photoreduction. The four spectra in each panel correspond to the different flavin redox states observed for yETF wild‐type (A), yETF‐βPhe19Tyr (B), hETF wild‐type (C), and hETF‐βTyr16Phe (D) in the course of the photoreduction: oxidized FAD (start spectrum, black line), anionic semiquinone (red line), reduced (blue line), and reoxidized FAD (green line).

**Figure 3:**
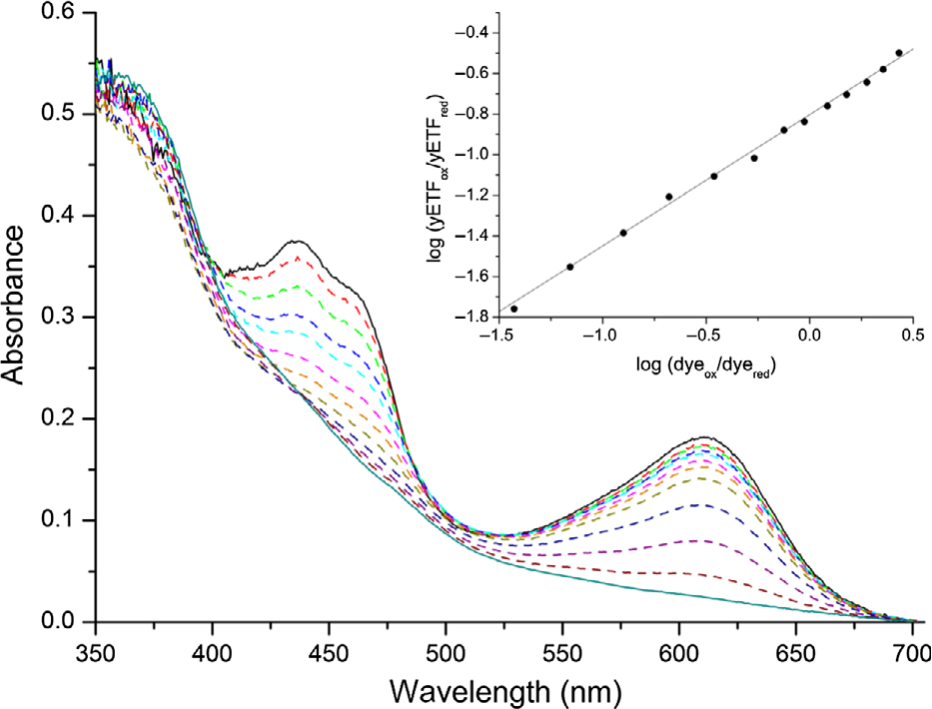
Determination of the redox potential of yETF wild‐type. Spectral changes in yETF (~ 25 μm) and indigo carmine (~ 20 μm; E°: −125 mV) observed in the course of the reduction reaction (2 h), indicating full reduction in the FAD cofactor and the dye and therefore a two‐electron transfer onto yETF. All measurements were carried out in 50 mm HEPES pH 7 + 1 mm DTT at a constant temperature of 25 °C. *Inset*, Nernst plot obtained from a single measurement by plotting the log(yETF_ox_/yETF_red_) as a function of the log(dye_ox_/dye_red_)—to determine the log(yETF_ox_/yETF_red_) and the log(dye_ox_/dye_red_) the relative absorption changes at 460 and 610 nm, respectively, were used. From the intercept a redox potential of −101 ± 2 mV was calculated (from four determinations).

Although all ETFs share the same cofactor, their electrochemical properties are tuned to the electron transfer pathway they service. Eukaryotic ETFs were found to efficiently stabilize the anionic flavin semiquinone state and therefore mainly receive single electrons from their donor systems 21, while the protein‐bound FAD cofactor in bacterial ETF from *Megasphera elsdenii* was shown to undergo two‐electron reduction when accepting electrons 8. To study the ability of yETF to stabilize the radical intermediate state, photoreduction and redox potential experiments were conducted. Upon illumination of oxidized yETF (Fig. 2A, black line) under anoxic conditions, a short‐lived anionic semiquinone (Fig. 2A, red line) was observed, which could easily be further reduced to the hydroquinone form (Fig. 2A, blue line). In the course of the reoxidation, no stable semiquinone intermediate was detected. When determining the redox potential of yETF, by applying the dye equilibration method 24, no stabilization of the flavin radical was observed, at all. The flavin chromophore and the reporting dye (indigo carmine; E°: −125 mV) were reduced almost synchronously (Fig. 3), allowing the generation of a Nernst plot by plotting the log(yETF_ox_/yETF_red_) as a function of the log(dye_ox_/dye_red_). From the intercept a midpoint potential of −101 ± 2 mV (oxidized to reduced) was calculated, which is more negative compared to eukaryotic ETFs, that is, around −20 mV 25, 26, but more positive than of the prokaryotic ETF from *Megasphera* (*Peptostreptococcus*)* elsdenii* (−259 mV) 27. Therefore, yETF can be considered a better electron acceptor than the ETF from *M*. *elsdenii*, but a weaker electron acceptor than the eukaryotic homologs.

### Comparison of the yeast ETF to the human homolog

**Figure 4:**
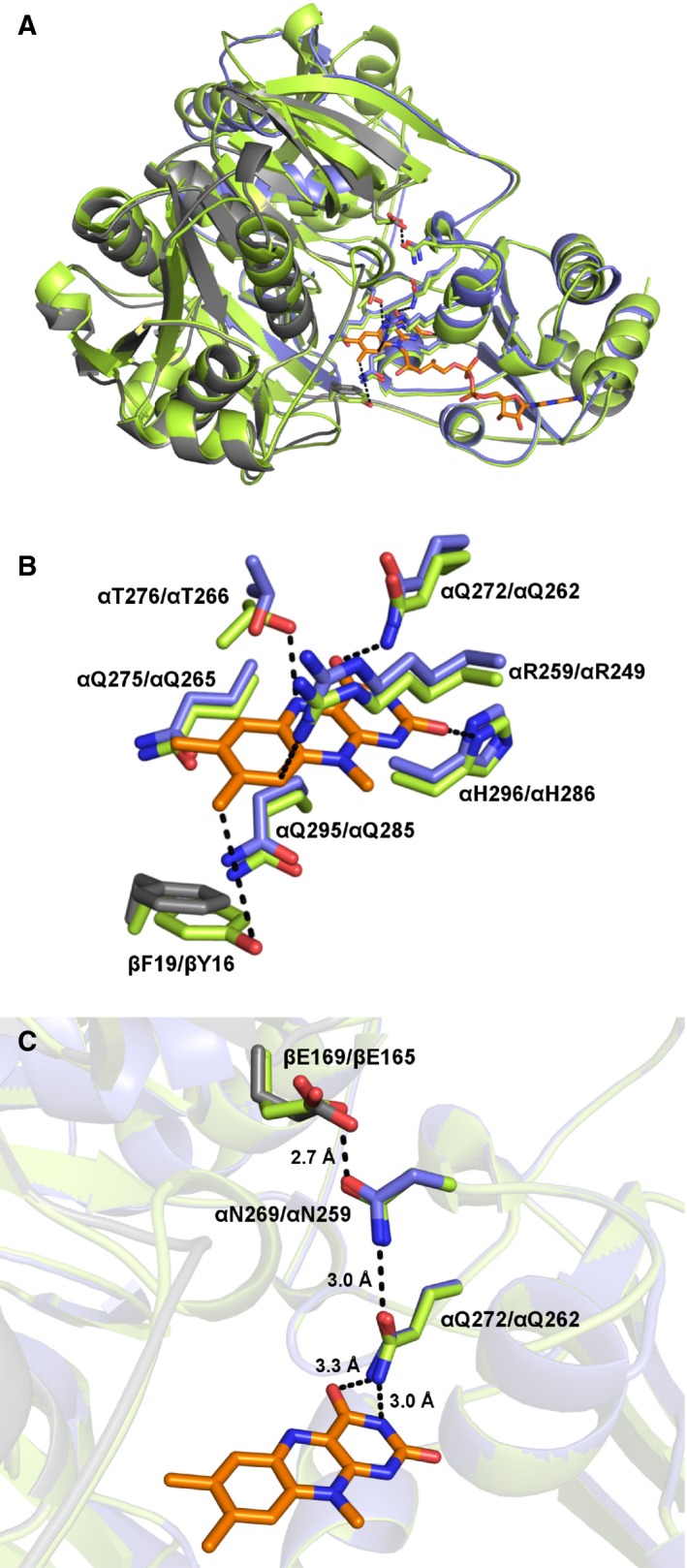
Structural comparison of yETF and hETF. (A) Overall structure comparison of yETF (blue: α‐subunit, gray: β‐subunit; model based on hETF) and hETF (green; PDB: 1EFV). (B) Comparison of the active site residues of yETF (blue: α‐subunit, grey: β‐subunit) and hETF (green). The FAD cofactor (orange) and the important amino acid residues are shown as sticks (blue and grey: yETF; green: hETF) and are labeled as follows: yETF/hETF. (C) Close‐up view of the well‐studied salt bridge formed between βGlu169 and αAsn269, which was shown to determine the dynamics of hETF (βGlu165 and αAsn259, respectively). The third residue displayed as sticks is αGln272, which is in H‐bonding distance to both αAsn269 and the N3‐C4=O locus of the flavin cofactor.

Based on the X‐ray structure of hETF, we have built a homology model of yETF indicating conservation of the overall fold (Fig. 4A) and composition of the amino acid residues forming the FAD‐binding pocket (Fig. 4B). The only obvious difference found in the active site of the model is the change of Tyr16 in hETF to Phe19 in yETF in the β‐subunit (Fig. 4B). Recently, we have shown that the FAD cofactor of hETF is oxidized at the 8α‐position to yield 8‐formyl‐FAD upon incubation under slightly alkaline conditions 17. Interestingly, this reaction is promoted by βTyr16 in hETF 17, 28, 29 and thus we hypothesized that yETF may be resistant toward oxidation of the FAD cofactor. To test our hypothesis, we transferred purified yETF to 50 mm HEPES pH 8.5 containing 1 mm DTT and incubated the sample at 25 °C for 24 h. After denaturation of the protein, the free cofactor was analyzed on HPLC‐DAD, which indeed revealed no 8‐formyl modification of the flavin cofactor. Instead, a mix of other flavin derivatives, which could not be identified due to their low abundance and insufficient purity, was found (Table 1).

**Table 1:** Cofactor modification in yETF wild‐type and variants after 24 h of incubation at pH 8.5: yETF wild‐type and the variants were diluted to a final concentration of ~ 50 μm using 50 mm HEPES pH 8.5 + 1 mm DTT and incubated at 25 °C for 24 h. After denaturation of the proteins their cofactor was analyzed with HPLC‐DAD. Total cofactor modification (%) refers to the amount of flavin cofactor altered upon incubation under alkaline conditions (column 2). Columns 3 and 4 provide a more detailed analysis of the obtained results—the fraction of flavin species that could be clearly identified as 8‐formyl‐FAD (based on retention time and flavin spectrum) is given in column 3, whereas the amount of unknown flavin species is summarized in column 4.

Protein	Total cofactor modification (%)	8f‐FAD (%)	Unknown modification (%)
WT	12	0	12
βF19Y	13	0	13
αN269A	26	26	0
βF19Y‐αN269A	42	31	11
βE169A	36	0	0
βF19Y‐βE169A	n.d.^a^	Yes^a^	n.d^a^

^a^As this variant is very unstable, cofactor modification cannot be assessed/quantified reliably.

In order to determine whether the tyrosine to phenylalanine replacement in yETF renders the 8α‐methyl group stable toward oxidation, we generated the hETF‐like variant yETF‐βPhe19Tyr. In the modification assay, however, this variant behaved rather similarly compared with wild‐type yETF by exhibiting an equal amount of overall flavin modification and not being able to form 8f‐FAD (Table 1). This finding, of course, raised the question which other factors are important for the oxidation reaction at this position. Since the cleavage of the salt bridge (between αAsn259 and βGlu165), which was shown to strongly influence the flexibility of the human protein 19, resulted in a much higher degree of formylation 17, we also generated two single variants (αAsn269Ala and βGlu169Ala) and two double variants (βPhe19Tyr‐αAsn269Ala and βPhe19Tyr‐βGlu169Ala) targeting this interaction (Fig. 4C). Although these variants exhibited a slightly higher degree of overall cofactor modification (Table 1), neither one of the variants showed 8α‐oxidation comparable with hETF.

Similarly, also the spectral properties remained almost unaffected by the amino acid replacements. Photoreduction of the single and the double variants, like for the wild‐type protein first yielded a short‐lived anionic semiquinone, which was further reduced to the flavin hydroquinone (Fig. 2B). This finding is in contrast to the homologous human proteins (wild‐type, αAsn259Ala and βGlu165Ala variant), which strongly stabilize the flavin semiquinone and, therefore, do not allow a full reduction in their FAD cofactor in the photoreduction experiment (Fig. 2C) 17.

### Biochemical characterization of Dld2

**Figure 5:**
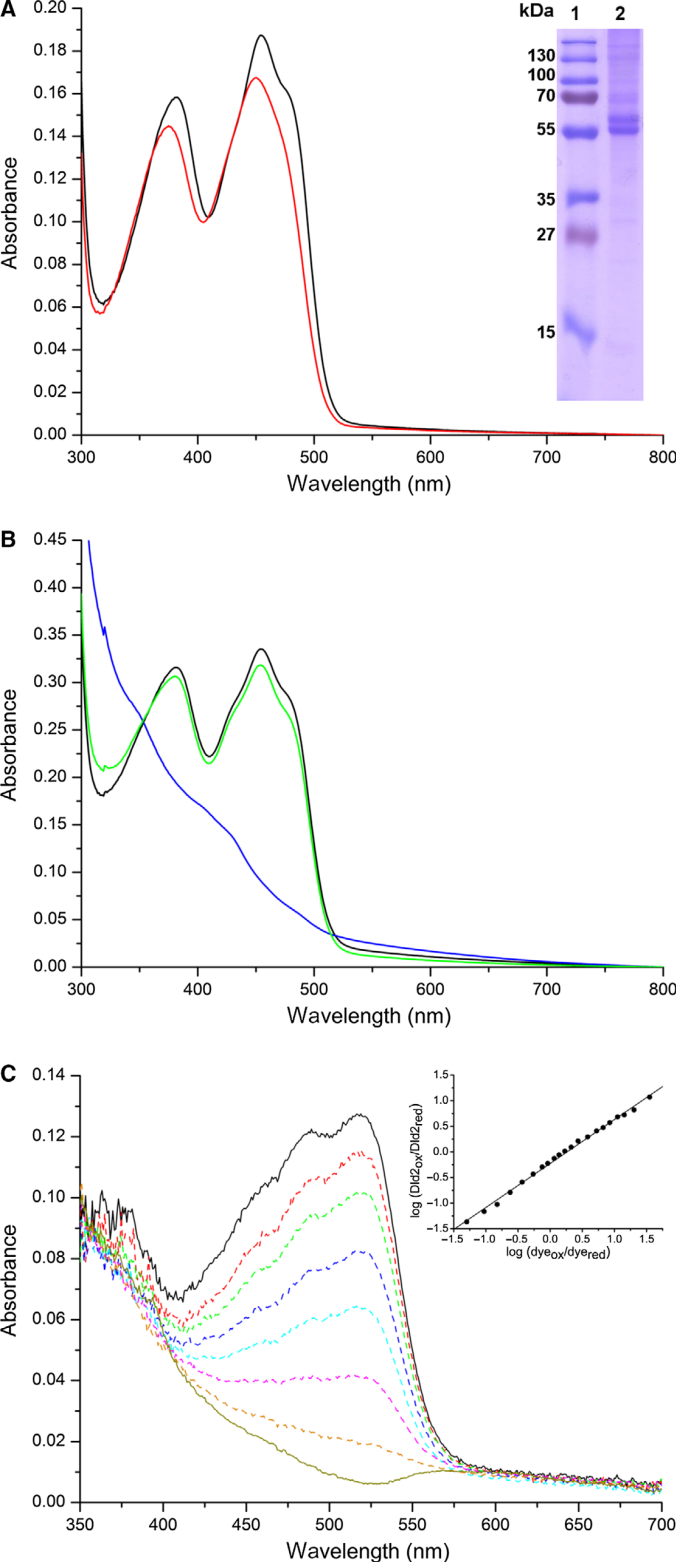
UV‐visible absorption spectrum and SDS/PAGE analysis (A), photoreduction (B) and determination of the redox potential (C) of Dld2. A, UV‐visible absorption spectra of Dld2 diluted to a final concentration of 10 μm with 50 mm HEPES, 150 mm NaCl pH 7 (black line) and denatured using 2% SDS (red line) were recorded between 300 and 800 nm at 25 °C. Based on the extinction coefficient of free FAD (denatured, red line) at 450 nm (11 300 m ^−1^ cm^−1^), the extinction coefficient of Dld2 at 450 nm (12 700 m ^−1^·cm^−1^) could be determined. Inset, SDS/PAGE analysis of a protein fraction containing pure Dld2. In lane 1 PageRuler^®^ prestained plus protein ladder (Thermo Fisher Scientific) and in lane 2 a protein fraction containing pure Dld2 is shown. The two bands correspond to nonprocessed Dld2 (α‐factor still attached, upper band) and to processed Dld2 (without α‐factor and possibly also without mitochondrial targeting sequence, lower band), respectively. (B) UV‐visible absorption spectra (300–800 nm) of oxidized (black line), fully reduced (blue line), and reoxidized (green line) Dld2 (diluted to a final concentration of 30 μm using 50 mm HEPES, 150 mm NaCl pH 7) recorded during anaerobic photoreduction. (C) Time‐dependent spectral changes of Dld2 (~ 10 μm) and phenosafranine (~ 10 μm; E°: −252 mV) observed in the course of the reduction reaction (25 min), indicating a full reduction in the FAD cofactor and the dye and therefore a two‐electron transfer onto Dld2. All measurements were carried out in 50 mm HEPES, 150 mm NaCl pH 7 at a constant temperature of 25 °C. Inset, Nernst plot obtained from a single measurement by plotting the log(Dld2_ox_/Dld2_red_) as a function of the log(dye_ox_/dye_red_)—to determine the log(Dld2_ox_/Dld2_red_) and the log(dye_ox_/dye_red_) the relative absorption changes at 450 and 520 nm, respectively, were used. From the intercept a redox potential of −246 ± 2 mV was calculated (from seven determinations).

To obtain satisfactory amounts of full‐length Dld2 from *S. cerevisiae*, recombinant expression was carried out in *K. phaffii*. Despite the presence of an α‐factor (secretion signal), the protein was not secreted to the culture medium, which required its purification from the cell pellet and yielded about 2–3 mg of pure enzyme per kg of wet cell weight. Owing to the incomplete processing of the secretion signal, purified Dld2 was isolated in two forms (Fig. 5A, inset), with and without the secretion signal (possibly also without targeting sequence). In its pure form, Dld2 exhibits absorption maxima at 383 and 454 nm, which are shifted to 375 and 450 nm, respectively, when the protein is denatured using 2% SDS, confirming the presence of an FAD cofactor (Fig. 5A).

To obtain information about the electrochemical properties of Dld2, we performed a photoreduction and determined the redox potential of the enzyme. Photochemical reduction in Dld2 required the use of high amounts of the redox mediators 5‐deaza‐FMN (2.5 μm) and methylviologen (6 μm) and yielded fully reduced enzyme after about half an hour of irradiation. Neither in the course of the reduction, nor upon reoxidation was a flavin radical observed (Fig. 5B), however, we found that under aerobic conditions Dld2 was reoxidized within a few seconds, which is in contrast to what one would expect from a dehydrogenase.

Like for yETF, the redox potential of Dld2 was determined by applying the xanthine/xanthine oxidase method first reported by Massey 24. The redox dye phenosafranine (E°: −252 mV) and the enzyme were reduced almost synchronously without the appearance of any radical intermediate species, indicating a two‐electron transfer onto Dld2 and the dye (Fig. 5C). This finding is in line with the photoreduction experiment and the slope near unity obtained when plotting the log of the relative absorption changes of Dld2 (450 nm) as a function of the log of the relative absorption changes in phenosafranine (520 nm). From the intercept, a redox potential of −246 ± 2 mV was calculated, which is clearly much more negative than that of yETF (midpoint potential: −101 mV) and thus in line with electron transfer from Dld2 to yETF.

### Kinetic properties of Dld2 and electron transfer to yETF

**Figure 6:**
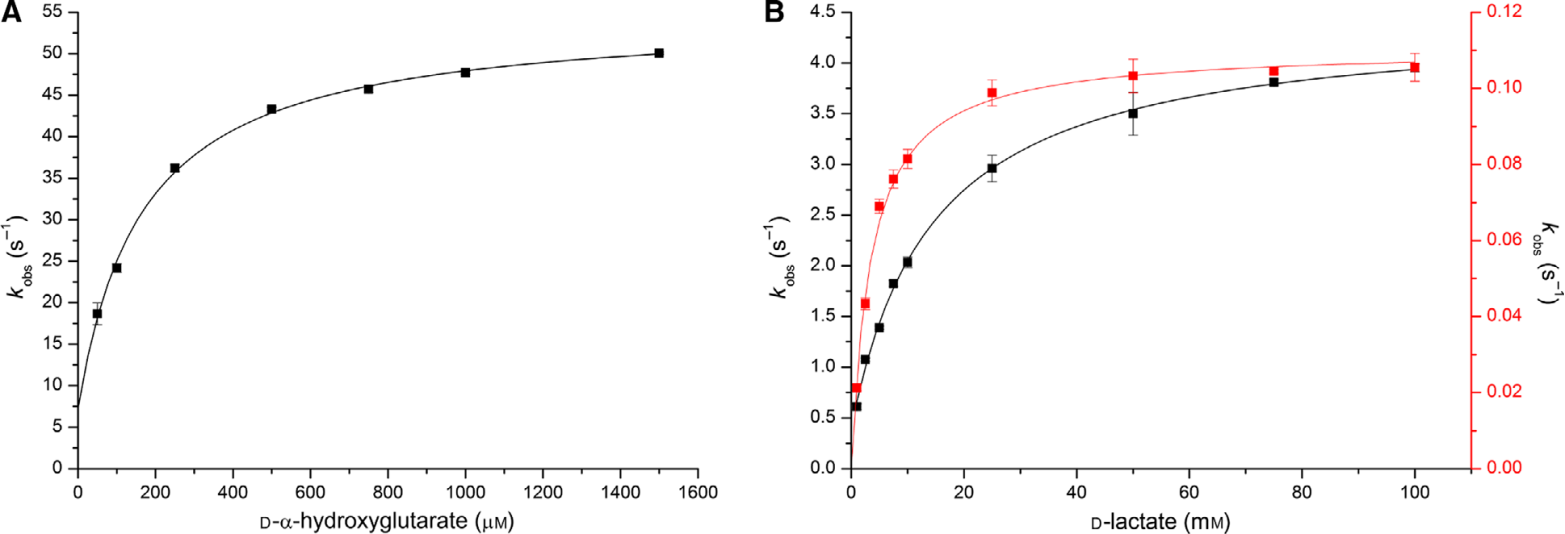
Rapid reaction kinetics of Dld2 using d‐α‐hydroxyglutarate (A) and d‐lactate (B) as substrate. The reductive half reaction of Dld2 was studied by mixing Dld2 (~ 10 μm final concentration) with varying concentrations of d‐α‐hydroxyglutarate (final concentrations: 50–1500 μm) and d‐lactate (final concentrations: 1–100 mm) in 50 mm HEPES, 150 mm NaCl pH 7 at 25 °C. The observed rate constants (*k*_obs_) were plotted as a function of the substrate concentration to give a hyperbolic curve that allowed the determination of the reductive rate (*k*_red_) and the dissociation constant (*K*_D_) of Dld2 with d‐α‐hydroxyglutarate (*K*_D_ = 170 ± 20 μm,* k*_red_ = 47 ± 1 s^−1^, observed rate constants at each substrate concentration were determined in triplicate; standard deviations are indicated by error bars) and d‐lactate (*K*_D_ = 15 ± 1 mm,* k*_red_ = 4.1 ± 0.1 s^−1^, observed rate constants at each substrate concentration were determined in triplicate), respectively. In the course of the reduction in Dld2 with d‐lactate a second slower phase was observed, which was analyzed in the same way to yield a *K*_D_ = 3.5 ± 0.2 mm and a *k*_red_ = 0.11 ± 0.00 s^−1^ (panel B, red line).

To learn more about the kinetic properties of full‐length Dld2 from *S. cerevisiae*, we conducted presteady‐state experiments in a stopped‐flow apparatus. By studying the rate of flavin reduction in the presence of different substrate concentrations (50–1500 μm d‐α‐hydroxyglutarate; 1–100 mm d‐lactate), observed rates were measured and plotted as a function of substrate concentration. Using a hyperbolic fit, limiting rate constants (*k*_red_) and dissociation constants (*K*_D_) for both substrates were determined (Fig. 6A,B, Table 2). The rate of reduction was 47 ± 1 s^−1^ and 4.1 ± 0.1 s^−1^ for d‐α‐hydroxyglutarate and d‐lactate with dissociation constants of 0.17 ± 0.02 mm and 15 ± 1 mm, respectively. Thus, d‐α‐hydroxyglutarate is oxidized more efficiently by the enzyme than d‐lactate. The graphs in Fig. 6A,B also indicate reversibility of the electron transfer reaction because the hyperbolic fit to the data yielded *y*‐axis intercepts of *k*_−1_ = 7.3 ± 1.7 s^−1^ and 0.4 ± 0.1 s^−1^ (~ 6% of *k*_red_) for d‐α‐hydroxyglutarate (~ 10% of *k*_red_) and d‐lactate, respectively.

**Table 2:** Kinetic parameters of Dld2 determined with d‐α‐hydroxyglutarate and d‐lactate under presteady‐state and steady‐state conditions. In the course of the presteady‐state experiment, 10 μm Dld2 (in reaction) was mixed with varying concentrations of d‐α‐hydroxyglutarate (final concentrations: 50–1500 μm) and d‐lactate (final concentrations: 1–100 mm) in 50 mm HEPES, 150 mm NaCl pH 7, and spectral changes were monitored for 1.5–150 s. From the absorbance changes at 450 nm observed rate constants were extracted and plotted as a function of the corresponding substrate concentrations to obtain reductive rates (*k*_red_) as well as dissociation constants (*K*_D_) for the interaction of Dld2 with both substrates. Kinetic characterization of Dld2 under steady‐state conditions was performed using 2,6‐dichlorophenol indophenol as a final electron acceptor. Dld2 (100 nm), 2,6‐dichlorophenol indophenol (125 μm), and varying final concentrations of d‐α‐hydroxyglutarate (25–1000 μm) or d‐lactate (1–250 mm) were mixed in 50 mm HEPES, 150 mm NaCl pH 7, and absorbance changes at 600 nm were recorded for 120 s. By plotting the initial rates as function of the substrate concentrations, additionally *k*_catapp_ and *K*_Mapp_ values for the interaction of Dld2 with both accepted substrates were obtained.

Kinetic parameters	d‐α‐Hydroxyglutarate	d‐Lactate
*k*_red_ (s^−1^)	47 ± 1	4.1 ± 0.1
*k*_−1_ (s^−1^)	7.3 ± 1.7	0.4 ± 0.1
*K*_D_ (mm)	0.17 ± 0.02	15 ± 1
*k*_catapp_ (s^−1^)	0.42 ± 0.02	1.2 ± 0.1
*K*_Mapp_ (mm)	0.35 ± 0.05	110 ± 10
*k*_catapp_ */K*_Mapp_ (m ^−1^·s^−1^)	1200 ± 300	11 ± 2

**Figure 7:**
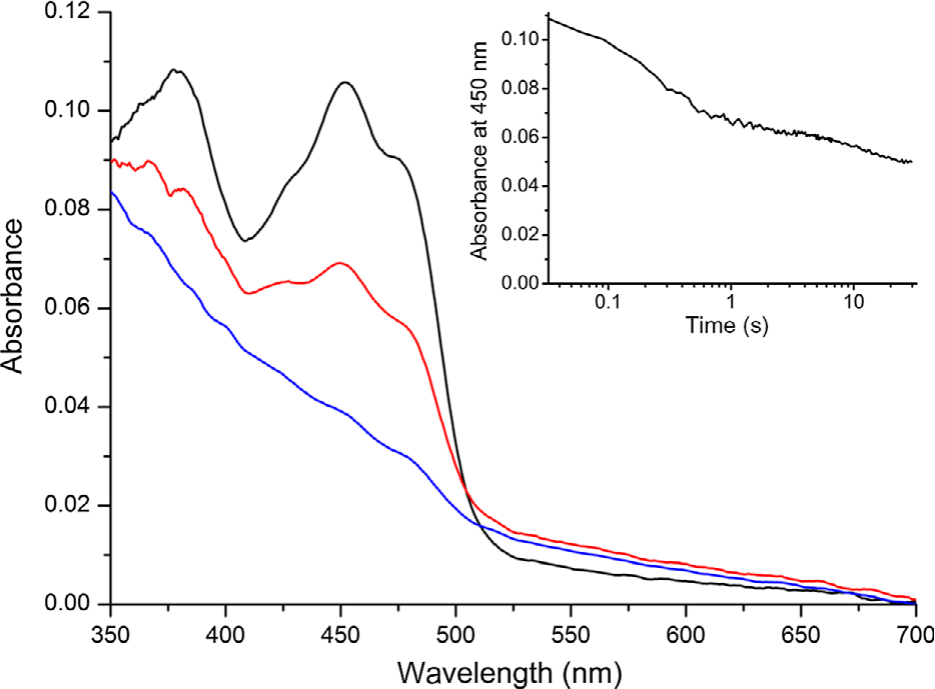
Spectral changes monitored in the course of the rapid reaction kinetics of Dld2 with d‐lactate. Spectral changes recorded within 3 s, when mixing Dld2 (final concentration: 10 μm) with d‐lactate (final concentration: 100 mm) in 50 mm HEPES, 150 mm NaCl pH 7. In a first, fast phase, oxidized Dld2 (black line) is partially reduced to yield the spectrum shown in red, before undergoing full reduction (blue line) in a second much slower phase. Inset, two distinct phases of flavin reduction observed in the course of the rapid reaction kinetics experiment involving Dld2 (final concentration: 10 μm) and d‐lactate (final concentration: 100 mm). Absorbance changes at 450 nm were plotted as a function of time (logarithmic time scale) to determine the rate of flavin reduction in both phases.

**Figure 8:**
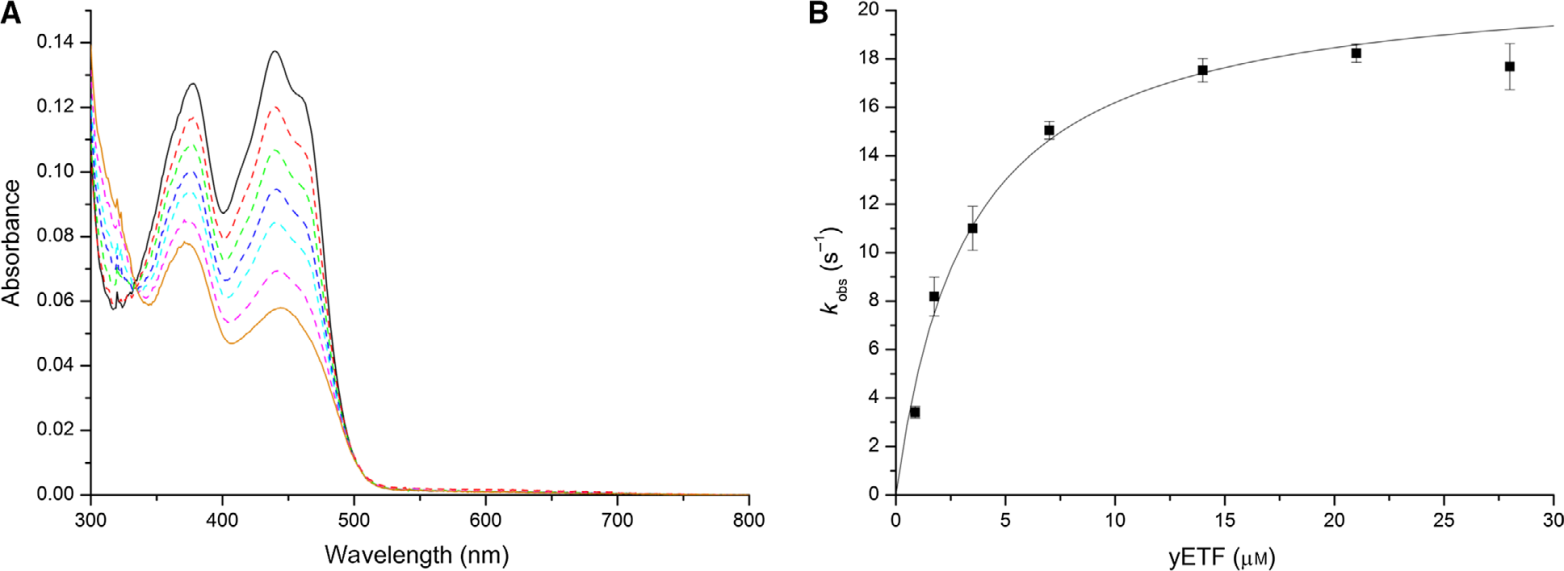
Electron transfer from Dld2 onto yETF. (A) yETF was diluted to a final concentration of 10 μm using 50 mm HEPES pH 7 + 1 mm DTT (black spectrum) and mixed with 1 mm d‐α‐hydroxyglutarate and 10 nm Dld2 at 25 °C. Then, time‐dependent spectral changes were monitored until no further changes were observed. The black spectrum corresponds to fully oxidized yETF, while the orange line represents the final spectrum recorded after 4 min. All other spectra recorded in the course of the reaction are shown using dashed lines. (B) Steady‐state turnover assays involving 1 mm d‐α‐hydroxyglutarate, 10 nm Dld2, yETF (0.875–28 μm) and 125 μm 2,6‐dichlorophenol indophenol as final electron acceptor were carried out in 50 mm HEPES pH 7 containing 1 mm DTT at 25 °C. Initial rates (normalized to enzyme concentration) were extracted from the absorbance changes recorded at 600 nm and plotted as a function of the yETF concentration. By applying a hyperbolic fit, the kinetic parameters *K*_Mapp_ and *k*_catapp_ were determined (*K*_Mapp_ = 3.3 ± 0.4 μm,* k*_catapp_ = 21 ± 1 s^−1^; initial rates at each yETF concentration were determined in triplicate; standard deviations are indicated by error bars).

Interestingly, a detailed analysis of the reduction reaction further revealed that flavin reduction proceeded in two distinct phases (Figs 6B and 7A,B). In the case of d‐α‐hydroxyglutarate the second phase was about 200 times slower than the first and thus exact quantification of the rate was not possible. In the case of d‐lactate, the second phase was determined to 0.11 ± 0.00 s^−1^ and exhibited a dissociation constant of 3.5 ± 0.2 mm. Next, we determined kinetic parameters for steady‐state conditions using 2,6‐dichlorophenol indophenol as an artificial electron acceptor under aerobic conditions. The turnover rates with d‐α‐hydroxyglutarate and d‐lactate were 0.42 ± 0.02 s^−1^ and 1.2 ± 0.1 s^−1^, respectively. However, the observed *K*_Mapp_ was much lower for d‐α‐hydroxyglutarate (350 ± 50 μm) than for d‐lactate (110 ± 10 mm) resulting in a higher catalytic efficiency for d‐α‐hydroxyglutarate (1200 ± 300 m ^−1^·s^−1^ vs 11 ± 2 m ^−1^·s^−1^). A summary of kinetic parameters is provided in Table 2. The large difference between the rate of reduction and the turnover rate suggested that the oxidative rate is rate limiting, which prompted us to analyze the rate of reoxidation of photoreduced and substrate‐reduced Dld2 using molecular oxygen as electron acceptor. Interestingly, photoreduced Dld2 was reoxidized about 5000 times faster than the enzyme reduced with d‐α‐hydroxyglutarate (*k*_ox_ = 3.3 × 10^4^ ± 0.2 × 10^4^ m ^−1^·s^−1^ vs 7 ± 1 m ^−1^·s^−1^). This result suggested that the reaction product (i.e., α‐ketoglutarate or pyruvate) tightly binds to the enzyme, thereby inhibiting the reoxidation by molecular oxygen. The addition of α‐ketoglutarate (or pyruvate) to photoreduced Dld2, either before or after the photoreduction, however, had no effect on the rate of reoxidation. Equally, titration of α‐ketoglutarate (or pyruvate) to oxidized Dld2 did not produce any changes in the UV‐visible absorption spectrum of the FAD cofactor, suggesting that the products only bind to the enzyme when generated in the course of the oxidation reaction. This interesting behavior prompted us to investigate the reoxidation of reduced Dld2 using its putative natural electron acceptor yETF. First, we incubated yETF (10 μm) with catalytic amounts of Dld2 (10 nm) and an excess of d‐α‐hydroxyglutarate (1 mm) and monitored the reduction in yETF by UV‐visible absorption spectrometry. As shown in Fig. 8A, yETF was reduced directly from the oxidized state to the fully reduced (hydroquinone) state within 4 min, which is in agreement with earlier experiments that have demonstrated the preferential two‐electron reduction in yETF.

For a more detailed kinetic characterization, steady‐state measurements using 2,6‐dichlorophenol indophenol as a final electron acceptor were performed (Fig. 8B, Table 3) yielding an apparent *k*_catapp_ of 21 ± 1 s^−1^ and *K*_Mapp_ of 3.3 ± 0.4 μm, which confirm an efficient electron transfer from Dld2 to yETF (catalytic efficiency: 6 × 10^6^ ± 1 × 10^6^ m ^−1^·s^−1^). Thus, electron transfer to the cognate electron acceptor, yETF, is several orders of magnitude faster than the reoxidation by either oxygen or 2,6‐dichlorophenol indophenol.

**Table 3:** Kinetic parameters determined for the electron transfer from Dld2 onto yETF under steady‐state conditions. The steady‐state parameters for the electron transfer were determined in a coupled assay involving 1 mm d‐α‐hydroxyglutarate, 10 nm Dld2, 125 μm 2,6‐dichlorophenol indophenol, and varying concentrations of yETF in 50 mm HEPES pH 7 containing 1 mm DTT (when using d‐lactate as substrate for Dld2 its reduction became rate limiting). By monitoring the decolorization of 2,6‐dichlorophenol indophenol at 600 nm for 120 s initial rates could be obtained and plotted as a function of the yETF concentrations to yield the kinetic parameters *k*_catapp_ and *K*_Mapp._

Kinetic parameters	d‐α‐Hydroxyglutarate	d‐Lactate
*k*_catapp_ (s^−1^)	21 ± 1	0.8 ± 0.4
*K*_Mapp_ (μm)	3.2 ± 0.4	Rate ETF‐concentration independent

## Discussion

### Biochemical characterization of yETF

Successful production of recombinant yETF and full‐length Dld2 from *S. cerevisiae* in *E. coli* and *K. phaffii*, respectively, enabled us to study their biochemical properties and the presumed electron transfer from Dld2 to yETF. Despite the extensive sequence similarity in the FAD‐binding pocket, we found that the properties of yETF diverge significantly from the human ortholog. The most salient differences concern the reduction behavior of yETF as evidenced by the absence of the anionic flavin semiquinone and the much more negative redox potential (−101 and −20 mV for yETF and hETF, respectively). Thus far, all ETFs characterized showed the preferential one‐electron reduction in FAD to yield the anionic semiquinone including mammalian and bacterial proteins 8, 17, 30, 31. In fact, the formation of the anionic semiquinone is thought to be critical for electron transfer from client dehydrogenases 32. Because of the obvious difference in the single active site residue provided by the β‐chain (βTyr16 in hETF and βPhe19 in yETF), we hypothesized that this amino acid exchange in yETF may be responsible for the distinct reduction behavior. In order to investigate this possibility, we generated the βTyr16Phe and βPhe19Tyr variant for hETF and yETF, respectively. While the replacement of βTyr16 by Phe in hETF strongly affected the stabilization of the anionic semiquinone, enabling full reduction of the protein in the course of the photoreduction experiment, the βPhe19Tyr variant of yETF did not behave like hETF (Fig. 2, panels A–D). Similarly, we have recently reported that replacement of βTyr16 by Phe impedes the oxidation of the 8α‐methyl to the 8‐formyl group and, therefore, we have also tested the propensity of wild‐type yETF and the βPhe19Tyr variant to form 8‐formyl‐FAD. Surprisingly, we have found that neither the wild‐type yETF nor the βPhe19Tyr variant formed 8‐formyl‐FAD. Even the introduction of further amino acid replacements (i.e., in the variants βPhe19Tyr‐αAsn269Ala and βPhe19Tyr‐βGlu169Ala) that significantly enhanced the rate of oxidation at the 8α‐methyl group in hETF wild‐type 17 did not result in increased formation of the 8‐formyl group. Thus, it appears that the redox properties of the FAD cofactor as well as the reactivity of the 8α‐methyl group is not simply governed by amino acid residues in the direct vicinity of the isoalloxazine ring but also by other factors such as amino acids in the second shell and the overall dynamics of the protein, which, for example, may influence the accessibility of the FAD cofactor. This view is in accordance with observations by Salazar *et al*. 18, who reported that the redox properties of ETFs are not solely affected by the amino acids found in the vicinity of the flavin moiety.

### Kinetic characterization of Dld2

Recently, the determination of steady‐state kinetic parameters by Becker‐Kettern *et al*. 16 revealed very slow turnover numbers for the oxidation of d‐lactate (1.3 s^−1^) and d‐α‐hydroxyglutarate (0.18 s^−1^) using N‐terminally truncated Dld2. Thus, we decided to reinvestigate the steady‐state kinetics of Dld2 using full‐length Dld2 recombinantly produced in *K. phaffii* (see Experimental procedures). However, we obtained very similar values for *k*_catapp_ for d‐lactate and d‐α‐hydroxyglutarate of 1.2 and 0.42 s^−1^, respectively, suggesting that the truncation at the N terminus does not compromise the catalytic properties of the enzyme. In order to identify the rate‐limiting step during turnover, we then investigated presteady‐state kinetics using stopped‐flow absorption spectrophotometry. This clearly showed that the reductive half reaction with d‐lactate and d‐α‐hydroxyglutarate proceeds at a limiting rate of 4.1 ± 0.1 s^−1^ and 47 ± 1 s^−1^, respectively (Table 2). In the case of d‐α‐hydroxyglutarate, the reductive rate is thus three orders of magnitude faster than the observed turnover. This result indicated that the oxidative half reaction is rate limiting. Surprisingly, when photoreduced Dld2 was reacted with dioxygen, we also found a rapid rate of reoxidation of 3.3 × 10^4^ ± 0.2 × 10^4^ m ^−1^·s^−1^. However, substrate‐reduced Dld2 reacted much slower with dioxygen. In the case of d‐α‐hydroxyglutarate, the rate of reoxidation was only 7 m ^−1^·s^−1^, that is, 5000 times slower as observed for the photoreduced enzyme. This suggested that the reaction product, pyruvate or α‐ketoglutarate, forms a complex with the reduced enzyme and thereby impedes reoxidation by dioxygen. Interestingly, the addition of either pyruvate or α‐ketoglutarate to photoreduced enzyme exhibited no effect on the rate of reoxidation with dioxygen. Thus, we conclude that during the oxidation of the substrate the formed product stabilizes a conformation of the enzyme in which access to the reduced FAD is denied. A similar behavior has been observed for other client enzymes of ETFs. In the case of acyl‐CoA dehydrogenases, reduced either by dithionite or by light irradiation, a very fast reoxidation was observed. If, however, the reaction product or a substrate/product mimic is bound to the protein, oxygen reactivity was reduced by a factor of 3000–4000 33, 34, 35. Wang and Thorpe 34 hypothesized that this effect is caused by the tight binding of enoyl‐CoA (and derivatives), which results in a significantly more hydrophobic active site and, therefore, prohibits the stabilization of superoxide radical species required for flavin reoxidation. Since α‐ketoglutarate and pyruvate are far less hydrophobic, it is difficult to imagine that oxygen reactivity is controlled by the same mechanism. Therefore, we suggest that product binding to Dld2 reduces the accessibility for oxygen to the active site and thereby impedes the reaction with the reduced FAD cofactor.

The occurrence of a product‐reduced enzyme complex is also supported by an increase in the long‐wavelength absorption (~ 510–625 nm) during the reductive half reaction (Fig. 7), indicating the formation of a charge‐transfer complex between α‐ketoglutarate or pyruvate and reduced Dld2. In keeping with this interpretation, we also observed that the long wavelength absorption disappeared during reoxidation of substrate‐reduced Dld2 with dioxygen. The formation of the charge‐transfer complex is apparently connected to the fast phase of reduction, which was also previously observed for acyl‐CoA dehydrogenases. It was postulated for the latter enzymes that formation of a charge‐transfer complex is indicative of tight product binding, which is used to shift the reaction equilibrium toward enoyl‐CoA formation 35, 36. Tight binding of the enoyl‐CoA product was argued to be necessary because the acyl‐CoA substrates have a very similar or even more positive redox potential than the FAD cofactor bound to the acyl‐CoA dehydrogenases, making acyl‐CoA oxidation rather unfavorable 36. Because the redox potential of the d‐lactate/pyruvate couple (−190 mV) is more positive than the midpoint potential of Dld2, it is plausible that charge‐transfer formation and tight product binding play a similar role in the yeast enzyme. In addition, the rather high *k*_−1_‐rates observed in the rapid reaction kinetics are in line with this hypothesis.

The slow reoxidation of substrate‐reduced Dld2 by oxygen and 2,6‐dichlorophenol indophenol prompted us to investigate reoxidation using the cognate electron acceptor, yETF. Initial experiments clearly demonstrated that yETF is rapidly reduced by catalytic amounts of Dld2 utilizing d‐α‐hydroxyglutarate as the substrate (Fig. 8A). Further steady‐state experiments established that the rate of electron transfer increased hyperbolically with the concentration of yETF, reaching a limiting value of 21 s^−1^ and thus approaching the rate of reduction in Dld2 with d‐α‐hydroxyglutarate. In other words, electron transfer from the product‐reduced enzyme complex to yETF is fast in contrast to reoxidation with oxygen or 2,6‐dichlorophenol indophenol and, therefore, product binding suppresses the undesired transfer of electrons to oxygen. The exact mechanism by which yETF obtains the electrons from the reduced enzyme is not clear yet but two scenarios are possible: (a) binding of yETF to the product‐reduced enzyme complex leads to the rapid release of the product and subsequently to electron transfer to yETF or (b) binding of yETF directly leads to electron transfer and to product dissociation from the reoxidized enzyme. As Dld2 very much behaves like acyl‐CoA dehydrogenases, it is tempting to speculate that also the mechanism of electron transfer to ETF is similar. In acyl‐CoA dehydrogenases the rapid reoxidation of the enzyme by hETF 33 was assumed to be promoted by a lower p*K*_a_ value of the reduced FAD cofactor in the product complex 37, 38, 39. In addition, tight product binding, as discussed above, shifts the equilibrium toward the reduced FAD cofactor and therefore electron transfer to ETF is more likely to occur prior to product release 37, 38, 39. Since Dld2 and the acyl‐CoA dehydrogenases are structurally very distinct, *p*‐cresol methylhydroxylase‐ (PCMH‐) fold vs acyl‐CoA dehydrogenase fold, and the chemical properties of their respective reaction products are rather different, further studies will be required to clearly resolve the exact mechanism of electron transfer from substrate‐reduced Dld2 to yETF.

### Two‐electron transfer from Dld2 to yETF

It should also be noted that reduction in yETF by Dld2 apparently involves the transfer of two electrons (Fig. 8A), which is in agreement with the large difference in redox potential of the two proteins (E° = −101 and −246 mV for yETF and Dld2, respectively). This finding is in line with our observations that yETF only weakly stabilizes the anionic semiquinone during photoreduction, and the reduction in Dld2, either by light irradiation or substrate, directly leads to the hydroquinone without the occurrence of a stable flavin semiquinone. This observation was rather surprising because all eukaryotic ETFs investigated so far were reported to operate through single electron transfer from the client dehydrogenase to the ETF 5, 8, 33, 40, 41. Furthermore, it was demonstrated that disproportionation of the flavin semiquinone to the oxidized and hydroquinone state occurs upon interaction with ETF‐QO, which is required for successful electron transfer 18, 40. Presently, the mechanism of electron transfer between yETF and its *in vivo* electron acceptor, Cir2p, is unknown. Previous studies involving various ETFs and their corresponding electron acceptors have indicated that electron transfer requires the transfer of single electrons and therefore relies on the stabilization of the flavin semiquinone 42, 43, 44. Owing to the electrochemical properties of yETF, it is thus conceivable that the mechanism of electron transfer to Cir2p is also different compared to the previously investigated electron transfer systems in eukaryotic cells.

## Experimental procedures

### Materials

All chemicals and media ingredients were purchased from Sigma‐Aldrich (St. Louis, MO, USA), Roth (Karlsruhe, Germany), Merck (Darmstadt, Germany), Fluka (Buchs, Switzerland) or Becton, Dickinson and Company (Franklin, Lakes, NJ, USA) and were of the highest grade commercially available.

All restriction enzymes used were ordered form Thermo Scientific/Fermentas (St. Leon‐Rot, Germany) or New England Biolabs (Ipswich, MA, USA) and Ni‐sepharose column material was obtained from GE Healthcare (Chalfont St. Giles, UK).

### Cloning and recombinant production of yETF (*E. coli*)

To produce the heterodimeric yETF in *E. coli* the respective gene was purchased from Thermo Scientific, codon optimized for the *E. coli* organism. The received gene (consisting of the coding sequence for the β‐subunit of the protein, of a 69 nucleotide noncoding linker sequence, and the coding sequence for the α‐subunit) was subcloned to the *E. coli* pJET vector and then, digested with *Nco*I and *Not*I to produce sticky ends on the DNA string. For gene expression the entire gene sequence subsequently was inserted into the vector pETM11‐His TEV, which added nucleotides coding for an N‐terminal hexahistidine tag to the gene sequence coding for the β‐subunit. Proper insertion of the gene was confirmed by sequencing, before transforming the recombinant plasmid with *E. coli* Rosetta (DE3)‐pLysS cells for gene expression.

For expression, LB medium containing 50 μg·mL^−1^ kanamycin as well as 20 μg·mL^−1^ chloramphenicol for selection was inoculated with an overnight culture to an optical density at 600 nm (OD_600_) of approximately 0.1. Then, cultures were left to grow to an OD_600_ of about 0.7 at 37 °C and 140 r.p.m., before adding 100 μm IPTG to induce gene expression. To maximize the protein yield, the cultures were incubated at 20 °C and 140 r.p.m. overnight.

Harvested cells were resuspended and lysed in binding buffer (50 mm HEPES, 15 mm imidazole, pH 7 + 1 mm DTT) by sonication with a Labsonic L sonication probe (B. Braun Biotech, Berlin, Germany) for 2 × 5 min. After centrifugation at 38 500 ***g*** for 1 h (4 °C), the cleared cell lysate was loaded onto a Ni‐nitrilotriacetic acid column equilibrated with binding buffer. After removing unspecifically bound protein with wash buffer (50 mm HEPES, 15 mm imidazole, pH 7 containing 1 mm DTT), yETF was eluted with elution buffer (50 mm HEPES, 500 mm imidazole, pH 7 containing 1 mm DTT). To confirm the presence of the desired protein in the fractions as well as to determine the quality of the purification SDS/PAGE analysis was used. Fractions containing yETF were pooled and dialyzed against 50 mm HEPES pH 7 + 1 mm DTT for 3 h. Then, the protein was concentrated to approximately 100 μm using Centripreps^®^ 30 kDa MWCO (Merck Millipore, Burlington, MA, USA), flash frozen with liquid nitrogen and stored at −80 °C until further use.

### Cloning and recombinant production of Dld2 (*K. phaffii*)

For recombinant expression of Dld2 in *K. phaffii* (formerly known *P. pastoris*), the respective gene was purchased from Thermo Scientific. On ordering, the sequence, flanked with the restriction sites *Xho*I (5′) and *Not*I (3′), was optimized for *K. phaffii*. Additionally, a nucleotide sequence coding for an octahistidine tag was added to the 3′‐end of the gene, to allow purification by Ni‐nitrilotriacetic acid affinity chromatography.

The received DNA string was cloned into the *K. phaffii* vector pPICZα, using the *Xho*I and *Not*I restriction sites and successful insertion was confirmed by sequencing. Having linearized the plasmid DNA using *Sac*I, electroporation was carried out, according to the instructions provided by the EasySelect™ ExpressionKit (Invitrogen, Carlsbad, CA, USA), to transform KM71H cells with our construct of interest. Additionally, the cells were transformed with pPICK‐PDI plasmid DNA to allow coexpression of the protein disulfide isomerase of *S. cerevisiae*.

Small‐scale expression in 96 well plates was performed as described by Weis *et al*. 45 in order to identify stably and well‐expressing clones. Clones, which displayed the high signal intensity when analyzing the expression supernatants with dot blot, were later used in large‐scale expression.

Large‐scale expression was performed in a BBI CT5‐2 fermenter (Sartorius, Göttingen, Germany) as described in 46. After an induction time of 24 h (a total of 250 g MeOH were added), the pH was set to 8 and cells and medium were separated by centrifugation at 3500 ***g*** for 30 min. As Dld2 was, despite the presence of an α‐factor, not properly secreted, the protein had to be purified from the cell pellets. Therefore, the pellets (cell wet weight: about 1.2–1.5 kg per fermenter) were suspended in an equal volume of lysis buffer (50 mm HEPES, 150 mm NaCl, 10 mm imidazole pH 8 + 2 mm DTT and 2 mm PMSF) and lysed using zirconium oxide beads in a Merkenschlager (Braun Biotech International, Melsungen, Germany).

After centrifugation of the suspension (38 500 ***g*** for 1 h), the cleared lysate was incubated with 15 mL of equilibrated Ni‐Sepharose™ (GE Healthcare) Fast Flow column material at 4 °C for approximately 1.5 h. Then, the supernatant was decanted and the column material was packed into an empty column and washed with approximately 150 mL wash buffer (50 mm HEPES, 150 mm NaCl, 20 mm imidazole, pH 8), before eluting Dld2 with elution buffer (50 mm HEPES, 150 mm NaCl, 300 mm imidazole, pH 8). SDS/PAGE was used to identify fractions containing the protein, which, subsequently were pooled and concentrated.

To obtain Dld2 of satisfactory purity a second purification step was needed, which required the buffer to be exchanged to 50 mm HEPES, 150 mm NaCl, pH 8 using a Sephadex G‐25 PD10 desalting column (GE Healthcare). Then, the protein solution was applied to a MonoQ™ 5/50 GL column (GE Healthcare), attached to an ÄKTA system (GE Healthcare) and equilibrated with buffer A (50 mm HEPES, 150 mm NaCl, pH 8). After a short wash with buffer A, the protein was eluted using a linear gradient of buffer A and buffer B (50 mm HEPES, 1 m NaCl, pH 8) (flow: 1 mL·min^−1^, linear gradient: 0 → 100% buffer B in 40 min). Fractions containing pure Dld2 (the protein eluted at approximately 40–55% buffer B) were pooled and concentrated using Centripreps^®^ 30 kDa MWCO (Merck Millipore). The concentrated protein was flash frozen using liquid nitrogen and stored at −80 °C until further use.

### Protein denaturation and determination of the extinction coefficient of yETF and Dld2

To determine the extinction coefficient of yETF, a spectrum of native as well as of denatured yETF was recorded as suggested by Macheroux 47. Assuming that the spectrum of the denatured protein equals the one of free FAD, the extinction coefficient of free FAD (11 300 m ^−1^·cm^−1^ at 450 nm) could be used to calculate the extinction coefficient of yETF at 450 nm (11 600 m ^−1^·cm^−1^) and 469 nm (9900 m ^−1^·cm^−1^; independent of flavin modification), respectively.

The extinction coefficient of Dld2 was determined as described for yETF. From the absorbance at 450 nm recorded for native and denatured enzyme, an extinction coefficient of 12 700 m ^−1^·cm^−1^ at 450 nm was calculated for Dld2.

### Photoreduction of yETF and Dld2

Photoreduction was performed under anoxic conditions as described by Massey *et al*. 48. For yETF, a solution containing 30 μm protein, 1 mm EDTA, 2 μm methylviologen and 1 μm 5‐deaza‐FMN was rendered anaerobic and transferred to a sealable quartz cuvette. After recording a UV‐visible absorption spectrum from 300 to 800 nm, the sample was irradiated and additional spectra were recorded at a constant temperature of 15 °C, until no further spectral changes were observed. Then, the lid was removed under aerobic conditions to initiate reoxidation of the protein, which again was monitored spectrophotometrically.

Photoreduction of Dld2 was performed as described for yETF, just the amount of EDTA (2.5 mm), 5‐deaza‐FMN (2.5 μm) and methylviologen (6 μm) was adapted, as the sample turned out to be difficult to reduce.

### Expression, purification and photoreduction of hETF‐βY16F

Cloning and expression of the hETF‐variant βY16F was carried out as described previously 17. Photoreduction was carried out as described for yETF (see above).

### Determination of the redox potential of yETF and Dld2

The redox potential of yETF was studied under anoxic conditions using a stopped‐flow device from Hi‐Tech (SF‐61DX2; TgK‐Scientific, Bradford‐on‐Avon, UK) placed in an anaerobic glove box (Belle Technology, Cambridge, UK) by applying the dye equilibrium method based on the xanthine/xanthine oxidase system, as reported by Massey 24. Two solutions, one containing ~ 50 μm yETF, 500 μm xanthine and 5 μm methyl viologen and a second one with ~ 40 μm indigo carmine (*A*_610_: ~ 0.5; E°: −125 mV) and ~ 200 nm xanthine oxidase, were prepared in 50 mm HEPES pH 7 + 1 mm DTT and mixed using the stopped‐flow device. Then, reduction in the flavin and the dye was monitored by recording 500 absorption spectra (350–700 nm) with a KinetaScan T diode array detector (MG‐6560) within 2 h (four measurements). From these data a Nernst plot was generated by plotting the log ([ox]/[red]) of the protein as a function of the log ([ox]/[red]) of the dye—to determine the log(yETF_ox_/yETF_red_) and the log(dye_ox_/dye_red_) the relative absorption changes at 460 and 610 nm, respectively, were used. Using the resulting intercept the redox potential of yETF could be determined as described by Minnaert 49.

To determine the redox potential of Dld2, two solutions, one containing ~ 20 μm Dld2, 500 μm xanthine and 5 μm methyl viologen and a second one with ~ 20 μm phenosafranin (*A*_520_: ~ 0.2; E°: −252 mV) and ~ 200 nm xanthine oxidase, were prepared in 50 mm HEPES, 150 mm NaCl pH 7 and mixed using the stopped‐flow device. Then, reduction in the flavin and the dye was monitored by recording 500 absorption spectra (350–700 nm) with a KinetaScan T diode array detector (MG‐6560) within 25 min (seven measurements). From these data, a Nernst plot was generated by plotting the log ([ox]/[red]) of the enzyme as a function of the log ([ox]/[red]) of the dye—to determine the log(Dld2_ox_/Dld2_red_) and the log(dye_ox_/dye_red_) the relative absorption changes at 450 and 520 nm, respectively, were used. Using the resulting intercept the redox potential of Dld2 could again be determined as described by Minnaert 49.

### Analysis of time‐dependent modification in yETF by HPLC

To analyze the degree and type of modification of the flavin bound to yETF, the protein was diluted to a concentration of approximately 50 μm at pH 8.5 and incubated at 25 °C for 24 h. Samples were taken after 0 and 24 h and inactivated by heat denaturation (10 min at 80–90 °C). After 10 min of centrifugation at 16 000 ***g***, the supernatant was transferred to HPLC vials for following HPLC‐DAD analysis with a Dionex Ultimate 3000 HPLC instrument (Thermo Fisher Scientific) equipped with an Atlantis^®^ dC18 column (5 μm, 4.6 × 250 mm; Waters, Milford, MA, USA) equilibrated with H_2_O/0.1% TFA, 7% acetonitrile. For all measurements, 100 μL of sample was injected and separation was carried out at a constant temperature of 25 °C and a flow rate of 1 mL·min^−1^ by applying the following gradient: 0–10 min, 7–9% acetonitrile; 10–25 min, 10% acetonitrile; 25–30 min, 12% acetonitrile; 30–32 min, 95% acetonitrile; 32–40 min, 7% acetonitrile. Elution of the different flavin species was monitored with a diode array detector (DAD; λ = 280, 370, 450, 460 nm, full spectrum).

### Homology modeling of yETF

A homology model of yETF was generated using the SWISS‐MODEL server 50, 51, 52. Since yETF was found to share the highest sequence coverage and identity with hETF (PDB: 1EFV 2) the crystal structure of the latter protein was chosen as template. Additionally, the flavin cofactor was modeled into the predicted structure of yETF by aligning the two proteins and copying the flavin moiety from hETF to the active site of the yeast homolog.

### Site directed mutagenesis

To get more information about the role of various active site residues found in yETF, variants were generated from the pETM11‐yETF wild‐type construct, using PCR‐based mutagenesis. All required nucleotide exchanges were introduced using forward and reverse primers carrying the desired mutations (for primers see Table 4). Constructs confirmed by sequencing were transformed with *E. coli* Rosetta (DE3)‐pLysS cells, to allow expression and purification as described for yETF wild‐type.

**Table 4:** Mutagenesis primers used for the generation of the various yETF variants, with the codon triplets carrying the desired mutations shown in bold.

Variant	Type	Primer sequence
βF19Y	Fwd.	5′–GCGTATTCTGGTTCCGGTTAAACGTGTTGTTGAT**TAT**CAGATTAAACCG–3′
	Rev.	5′–CGGTCAGGGTTTTATTCACACGCGGTTTAATCTG**ATA**ATCAACAACACG–3′
αN269A	Fwd.	5′–CGTGCAAGCGTTGATAATGGCCTGTGTGAT**GCT**AGCCTGCAGATTGG–3′
	Rev.	5′–GCAACAACTTTACCGGTCTGACCAATCTGCAGGCT**AGC**ATCACACAGG–3′
βE169A	Fwd.	5′–CTGGATAATGGTCGTGTTCAGGTTACCCGT**GCA**ATCGATGATGGTG–3′
	Rev.	5′–GGCTTGCTTCAATAACTTCTTCACCATCATCGAT**TGC**ACGGGTAACC–3′

### Reductive and oxidative half reaction (Dld2)

To study the presteady‐state kinetics of Dld2, time‐dependent spectral changes in the flavin absorption were recorded under anoxic conditions using a stopped‐flow device (Hi‐Tech, TgK Scientific), placed in a glove box (Belle Technology), and monitored with a KinetikaScan T diode array detector (MG‐6560). The following analysis was performed by fitting the data points recorded at 450 nm with the kinetic studio Software (TgK Scientific). For the determination of the reductive rate of Dld2, its flavin reduction was studied in the presence of seven different d‐α‐hydroxyglutarate (final concentration: 50–1500 μm) and nine d‐lactate (final concentration: 1–100 mm) concentrations. A 20 μm enzyme solution and the substrate solutions were prepared in 50 mm HEPES, 150 mm NaCl pH 7, before mixing them in the stopped‐flow device and recording the spectral changes between 350 and 700 nm (measurements were performed four times at each substrate concentration). The extracted observed rate constants were plotted as a function of the respective substrate concentrations to obtain a hyperbolic curve, which allowed determination of the reduction rates (*k*_red_) as well as of the dissociation constants (*K*_D_).

To study the effect of product binding on the rate or reoxidation, one sample of reduced Dld2 (20 μm) was generated using photoreduction (see above) and a second one by adding about 1.5 eq of d‐α‐hydroxyglutarate. Then, both samples were mixed with air saturated buffer (20 °C) in a stopped‐flow device and spectral changes between 350 and 700 nm were monitored for 1.5–3 s and 25–50 min, respectively. To obtain bimolecular reoxidation rates, the observed rate constants were divided by the final oxygen concentration in the reaction mixtures (final concentration: 140 μm).

### Qualitative analysis of the electron transfer from Dld2 to yETF

To analyze the possible electron transfer from Dld2 to yETF, yETF was diluted to a final concentration of ~ 10 μm using 50 mm HEPES pH 7 + 1 mm DTT and mixed with 1 mm of d‐α‐hydroxyglutarate. After recording an initial UV‐visible absorption spectrum between 300 and 800 nm, Dld2 was added to a final concentration of 10 nm and changes in the absorption characteristics of yETF were monitored over time.

### Steady‐state kinetics (Dld2)

To study the kinetic parameters of Dld2 under steady‐state conditions, a coupled assay with 2,6‐dichlorophenol indophenol as final electron acceptor was used. Solutions containing varying final concentrations of d‐α‐hydroxyglutarate (25–1000 μm) or d‐lactate (1–250 mm) were prepared in 50 mm HEPES, 150 mm NaCl pH 7 and mixed with 125 μm 2,6‐dichlorophenol indophenol. After 10 min of incubation at 25 °C, Dld2 was added to a final concentration of 100 nm and absorbance changes at 600 nm were recorded for 120 s. By plotting the extracted initial rates as a function of the corresponding substrate concentrations the kinetic parameters *K*_Mapp_ and *k*_catapp_ could be determined.

### Steady‐state analysis of the electron transfer from Dld2 onto yETF

Steady‐state parameters for the electron transfer of Dld2 onto wild‐type yETF were determined by performing a coupled assay with 2,6‐dichlorophenol indophenol as final electron acceptor. Therefore, buffer (50 mm HEPES pH 7 containing 1 mm DTT), Dld2 (10 nm), yETF (0.875–28 μm) and 2,6‐dichlorophenol indophenol (125 μm) were mixed and incubated at 25 °C for 10 min. Then, the reaction was started by adding 1 mm d‐α‐hydroxyglutarate, the substrate of Dld2, and the decrease in absorption at 600 nm was monitored at 25 °C for 120 s (measurements at each yETF concentration were performed in triplicate). To determine the kinetic parameters, *K*_Mapp_ and *k*_catapp_, the extracted initial velocities (normalized to enzyme concentration) were plotted as a function of the respective yETF concentrations yielding a hyperbolic curve, which was fitted using the program origin 7 (OriginLab, Northampton, MA, USA).

### Titration of yETF and Dld2 with α‐ketoglutarate

To study the binding of α‐ketoglutarate to yETF and Dld2, 800 μL of protein diluted to a final concentration of about 10 μm using 50 mm HEPES pH 7 + 1 mm DTT and 50 mm HEPES, 150 mm NaCl pH 7, respectively, were transferred to a quartz cuvette (reference just 800 μL of buffer) and a UV‐visible absorption spectrum between 300 and 800 nm was recorded. Then, 10‐μL aliquots of 2.5 mm α‐ketoglutarate were added to both the reference and the measurement cuvette and further spectra were recorded after an incubation time of 1 min following the addition of the metabolite.

## Conflict of interest

The authors declare no conflict of interest.

## Author contributions

MT, JB, and CRT prepared the constructs for heterologous gene expression; MT and JB expressed the genes, purified the produced proteins, and performed biochemical experiments; MT, JB, and PM designed the biochemical and kinetic experiments and interpreted the data and wrote the manuscript.
